# Association of acetaminophen use with perinatal outcomes among pregnant women: a retrospective cohort study with propensity score matching

**DOI:** 10.1186/s12884-024-06480-5

**Published:** 2024-04-11

**Authors:** Man Xu, Ran Wang, Boran Du, Ying Zhang, Xin Feng

**Affiliations:** 1 Department of Pharmacy, Beijing Obstetrics and Gynecology Hospital, Capital Medical University, No.17, Qi He Lou Street, Dongcheng District, Beijing, 100010 China; 2 Department of Clinical Pharmacology, College of Pharmaceutical Sciences, Capital Medical University, Beijing, China; 3 Laboratory for Clinical Medicine, Capital Medical University, Beijing, China

**Keywords:** Acetaminophen, Pregnant women, Newborns, Pregnancy outcomes

## Abstract

**Background:**

Although acetaminophen is widely used in women during pregnancy, its safety has not been clearly stated. The study aimed to investigate the association between acetaminophen use and adverse pregnancy outcomes in pregnant women in China.

**Methods:**

We conducted a retrospective cohort study by collecting data on pregnant women who delivered in the Beijing Obstetrics and Gynecology Hospital from January 2018 to September 2023. An acetaminophen use group and a control group were formed based on prenatal exposure to acetaminophen. The pregnancy outcomes that we focused on were stillbirth, miscarriage, preterm birth, APGAR score, birth weight, and congenital disabilities. Pregnant women exposed to acetaminophen were matched to unexposed in a 1:1 ratio with propensity score matching, using the greedy matching macro. SPSS software was used for statistical analysis. Multivariable logistics regression was used to assess the association between acetaminophen use during pregnancy and adverse pregnancy outcomes.

**Results:**

A total of 41,440 pregnant women were included, of whom 501 were exposed to acetaminophen during pregnancy, and 40,939 were not exposed. After the propensity score matching, the acetaminophen use and control groups consisted of 501 pregnant women each. The primary analysis showed that acetaminophen exposure during pregnancy was associated with an increased risk of stillbirth (adjusted OR (aOR) = 2.29, 95% CI, 1.19–4.43), APGAR score < 7 at 1 min (aOR = 3.28, 95% CI, 1.73–6.21), APGAR score < 7 at 5 min (aOR = 3.54, 95% CI, 1.74–7.20), APGAR score < 7 at 10 min (aOR = 3.18, 95% CI, 1.58–6.41), and high birth weight (HBW) (aOR = 1.75, 95% CI, 1.05–2.92). Drug exposure during the first and second trimesters increased the odds of stillbirth, miscarriage, APGAR < 7, and the occurrence of at least one adverse pregnancy outcome. In addition, the frequency of drug use more than two times was associated with a higher risk of preterm birth and APGAR score < 7.

**Conclusions:**

Exposure to acetaminophen during pregnancy was significantly associated with the occurrence of adverse pregnancy outcomes, particularly exposure in the first and second trimesters and frequency of use more than twice. It is suggested that acetaminophen should be prescribed with caution in pregnant women.

**Supplementary Information:**

The online version contains supplementary material available at 10.1186/s12884-024-06480-5.

## Background

Acetaminophen is an over-the-counter medication that reduces fever and relieves mild to moderate pain [1]. It is widely used by pregnant women, with usage rates reaching 65.1% [2]. However, acetaminophen should be used with caution due to potential toxicity to both the mother and fetus.

For the sake of ethics, randomized controlled clinical trials could not be conducted on pregnant women to evaluate the safety of acetaminophen use [3]. Therefore, the safety of using acetaminophen during pregnancy was mainly assessed by cohort or case-control studies. A growing body of research has shed light on the potential association between acetaminophen use and adverse pregnancy outcomes, while the findings remain controversial [4, 5].

It is worth noting that certain studies did not consider the impact of dose and duration of acetaminophen exposure on adverse outcomes. Although, these studies have suggested that exposure to acetaminophen during pregnancy was linked to an increased risk of adverse pregnancy outcomes [4]. Acetaminophen could cross the placenta and blood–brain barrier, and its metabolism may be changed during pregnancy, which would make pregnant women and their fetuses more vulnerable to toxic effects [6]. Different levels of acetaminophen accumulated in the body might affect the liver morphology of the fetus. The hypothesis suggested that harm to the fetal liver might reduce blood stem cells reaching vital organs, consequently increasing the risk of growth and development [7].

Considering the widespread use and the potential risks of acetaminophen in pregnant women, there is an urgent need to study the association between acetaminophen use during pregnancy and the potential dangers of pregnancy. Our study aimed to explore the risks associated with acetaminophen use among pregnant women in China. Furthermore, we attached importance to the drug use in different trimesters of pregnancy and the dose on pregnancy outcomes, which is crucial to provide evidence-based support for the safe use of acetaminophen during pregnancy in clinical settings.

## Methods

### Study design and ethics

A retrospective cohort study was conducted using data from the electronic medical record (EMR) system of the Beijing Obstetrics and Gynecology Hospital Affiliated with Capital Medical University from January 2018 to September 2023. The Beijing Obstetrics and Gynecology Hospital is a tertiary healthcare facility that serves multiple public health functions of provincial maternal and child healthcare hospitals. The patient EMR system has a unique code to support the inquiry and tracking of patient medical records and medication information. Permission for data analysis was approved by the Ethics Committee of Beijing Obstetrics and Gynecology Hospital (No. 2022-KY-057), and written informed consent was obtained from all patients.

The study enrolled pregnant women who met the following criteria: (1) those who had a record of delivery or termination of pregnancy in our hospital; (2) for women with multiple births during the study period, only data from their latest pregnancy was included; and (3) those who were 18 years of age or older. The exclusion criteria for the study were: (1) incomplete information, (2) known cause of congenital disabilities (e.g., chromosomal or genetic disease), (3) use of teratogenic drugs during pregnancy, and (4) pregnant women with multiple pregnancies.

The exposure and control groups were formed from the included samples based on whether they were exposed to acetaminophen during pregnancy. The exposure group consisted of pregnant women who had been exposed to acetaminophen during pregnancy, regardless of the dosage and course of the treatment. The control group consisted of pregnant women who had not been exposed to acetaminophen or any other medication during pregnancy.

### Data collection and outcome measures

Diagnostic information, medication data, and perinatal outcomes were extracted from the EMR. The drug information collected included: acetaminophen (dose, course, frequency, and time of treatment), non-steroidal anti-inflammatory drugs (NSAIDs), antibiotics, antiviral drugs, and Chinese patent drugs for the common cold. Perinatal outcomes included preterm birth (< 37 gestation weeks) [8], stillbirth [9], miscarriage (spontaneous abortion) [10], APGAR score (1, 5, and 10 min) [11], low birth weight (LBW) (< 2500 g) [12], high birth weight (HBW) (≥ 4000) g) [13], and congenital disabilities.

### Confounding factors

Adjustments for confounding factors included maternal age, pre-pregnancy Body Mass Index (BMI) (Underweight, Normal weight, Overweight, and Obese), number of previous pregnancies (0 = 1,1 = ≥ 2), previous live births (0 = 0, 1 = ≥ 1), history of adverse perinatal outcomes (0 = No, 1 = Yes), type of labor (Spontaneous, Caesarean section, Induced), Infection with COVID-19 (0 = No, 1 = Yes), comorbidities (fever, common cold, upper respiratory tract infection, immuno-associated diseases, hypertensive disorders, and preexisting and gestational diabetes) (0 = No, 1 = Yes) and co-medication (NSAIDs, antibiotics, antiviral drugs, Chinese patent drugs for the common cold) (0 = No, 1 = Yes).

### Statistical analysis

The descriptive analyses were conducted to describe the characteristics of pregnant women in the acetaminophen exposure and control groups. The Kolmogorov-Smirnov test was used to test the normality of continuous variables. The continuous variables that conformed to a normal distribution were expressed as mean ± standard deviation (SD), and those that did not conform to a normal distribution were expressed as median and interquartile range (IQR). The categorical data were expressed as frequencies. Parametric Student’s t-test or non-parametric Mann-Whitney test was applied to compare continuous variables. The chi-square test or Fisher’s exact test was used to compare categorical variables.

We used a propensity score to estimate the impact of group accounting for confounding by covariates. The propensity score was calculated by fitting a logistic regression model including maternal age, pre-pregnancy BMI, number of previous pregnancies, previous live births, history of adverse perinatal outcomes, type of labor, Infection with COVID-19, comorbidities, and co-medication. Pregnant women exposed to acetaminophen were matched to unexposed in a 1:1 ratio with propensity score matching, using the greedy matching macro.

The association between acetaminophen use and adverse perinatal outcomes was estimated by logistic regression, with odds ratios (OR) and 95% confidence intervals (CIs). The Hosmer–Lemeshow test was used to assess the goodness of fit of the model. Primarily, we compared acetaminophen exposure and control groups to investigate the association between drug exposure and adverse perinatal outcomes. Then, we conducted a stratified analysis by categorizing the samples based on the frequency and time of acetaminophen use. *P*-values < 0.05 were considered statistically significant, and all tests were two-sided. The statistical analysis was carried out using SPSS software (version 24.0).

## Results

**Fig. 1:**
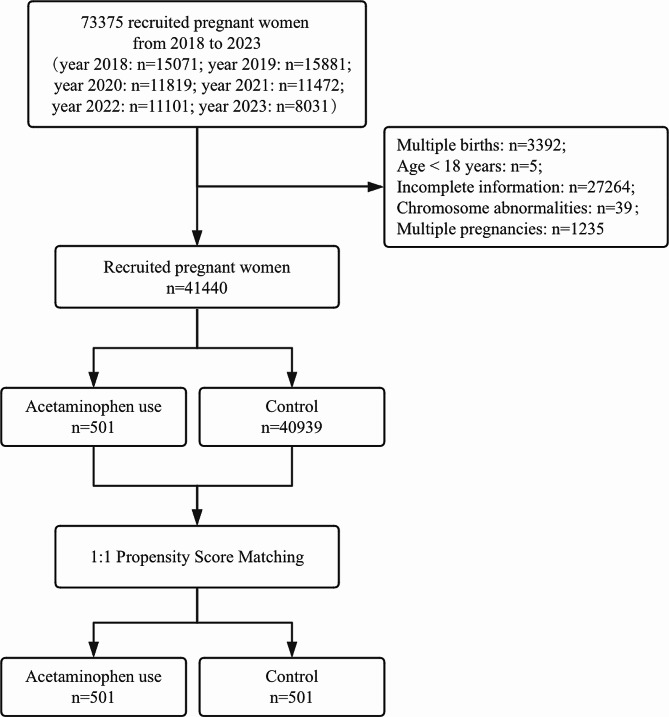
Flow diagram showing the selection process of the exposure and control groups included in the study

From January 2018 to September 2023, 73,375 pregnant women had a record of delivery or termination of pregnancy in the Beijing Obstetrics and Gynecology Hospital. After screening, 41,440 pregnant women were eventually included, with 501 exposed to acetaminophen during pregnancy and 40,939 not exposed (Fig. 1). Before propensity score matching, most variables had statistically significant differences between the exposed and control groups. Then, we included 1002 patients after propensity score matching at a 1:1 ratio (Match Tolerance = 0.02), and all variables were well-balanced (Table 1).

**Table 1:** Characteristics of the study population with or without acetaminophen use during prenancy

Characteristics	Before matching (*n* = 41,440)			After matching (*n* = 1002)		
	Acetaminophen use (*n* = 501)	Control (*n* = 40,939)	*p* value	Acetaminophen use (*n* = 501)	Control (*n* = 501)	*p* value
**Maternal age (median and IQR)**	32 (30–35)	33 (30–35)	0.005	32 (30–35)	32 (30–35)	0.991
**Pre-pregnancy BMI, kg/m** ^**2**^ **n (%)**			0.003			0.158
Underweight (< 18.5)	18 (3.59)	2124 (5.19)		18 (3.59)	7 (1.40)	
Normal weight (18.5–24.9)	211 (42.12)	14,196 (34.68)		211 (42.12)	209 (41.72)	
Overweight (25.0−29.9)	116 (23.15)	9733 (23.77)		116 (23.15)	124 (24.75)	
Obese (≥ 30.0)	156 (31.14)	14,886 (36.36)		156 (31.14)	161 (32.13)	
**Number of previous pregnancies (median and IQR)**	1(0–2)	1(0–2)	< 0.001	1(0–2)	1(0–2)	0.779
1	187 (37.33)	10,699 (26.13)		187 (37.33)	176 (35.13)	
≥ 2	314 (62.67)	30,240 (73.87)		314 (62.67)	325 (64.87)	
**Previous live births (median and IQR)**	0(0–1)	1(0–1)	< 0.001	0(0–1)	0(0–1)	0.820
0	281 (56.09)	16,426 (40.12)		281 (56.09)	282 (56.29)	
≥ 1	220 (43.91)	24,513 (59.88)		220 (43.91)	219 (43.71)	
**History of adverse pregnancy outcomes n (%)**			0.063			0.250
Yes	68 (13.57)	4485 (10.96)		68 (13.57)	56 (11.18)	
No	433 (86.43)	36,454 (89.04)		433 (86.43)	445 (88.82)	
**Type of labour n (%)**			< 0.001			0.955
Spontaneous	224 (44.71)	22,282 (54.43)		224 (44.71)	222 (44.31)	
Caesarean section	254 (50.70)	16,478 (40.25)		254 (50.70)	254 (50.70)	
Induced	23 (4.59)	2179 (5.32)		23 (4.59)	25 (4.99)	
**Infection with COVID−19 n (%)**			< 0.001			0.446
Yes	25 (4.99)	308 (0.75)		25 (4.99)	20 (3.99)	
No	476 (95.01)	40,631 (99.25)		476 (95.01)	481 (96.01)	
Comorbidities* **n (%)**			< 0.001			0.558
Yes	314 (62.67)	10,477 (25.59)		314 (62.67)	305 (60.88)	
No	187 (37.33)	30,462 (74.41)		187 (37.33)	196 (39.12)	
**Co-medication** ^#^ **n (%)**			< 0.001			0.335
Yes	357 (71.26)	16,170 (39.50)		357 (71.26)	343 (68.46)	
No	144 (28.74)	24,769 (60.50)		144 (28.74)	158 (31.54)	

All data were expressed as the median and IQR or n (%)

Abbreviations: PS, propensity score; IQR, interquartile range; SD, standard deviation; BMI, Body Mass Index; COVID-19, the coronavirus disease 2019

* Comorbidities during pregnancy included fever, common cold, upper respiratory tract infection, immuno-associated diseases, hypertensive disorders, and preexisting and gestational diabetes

^#^ Co-medication during pregnancy included NSAIDs, antibiotics, antiviral drugs, Chinese patent drugs for the common cold

As shown in Table 2, acetaminophen use during pregnancy was associated with an increased odds of stillbirth (adjusted OR (aOR) = 2.29, 95% CI, 1.19–4.43), APGAR score < 7 at 1 min (aOR = 3.28, 95% CI, 1.73–6.21), APGAR score < 7 at 5 min (aOR = 3.54, 95% CI, 1.74–7.20), APGAR score < 7 at 10 min (aOR = 3.18, 95% CI, 1.58–6.41), and HBW (aOR = 1.75, 95% CI, 1.05–2.92) compared to the control group. Our results demonstrated consistency after conducting a crude analysis and adjusting for confounding factors. However, there was no significant association between acetaminophen use during pregnancy and miscarriage, preterm birth, LBW, congenital disabilities, and at least one outcome.

**Table 2:** Exposure to acetaminophen during pregnancy and risk of adverse perinatal outcomes in comparison with control group

Outcomes	Control (*n* = 501) n (%)	Acetaminophen use (*n* = 501) n (%)	Crude OR (95% CI)	*Adjusted OR (95% CI)
**Stillbirth**				
Yes	24 (4.8)	41 (8.2)	1.77 (1.05–2.98)	2.29 (1.19–4.43)
No	477 (95.2)	460 (91.8)	1.00	1.00
**Miscarriage**				
Yes	24 (4.8)	36 (7.2)	1.54 (0.90–2.62)	1.83 (0.93–3.58)
No	477 (95.2)	465 (92.8)	1.00	1.00
**Preterm birth**				
Yes	42 (8.4)	47 (9.4)	1.13 (0.73–1.75)	1.07 (0.68–1.68)
No	459 (91.6)	454 (90.6)	1.00	1.00
**APGAR score at 1 min**				
< 7	20 (4.0)	47 (9.4)	2.49 (1.45–4.27)	3.28 (1.73–6.21)
Normal	481 (96.0)	454 (90.6)	1.00	1.00
**APGAR score at 5 min**				
< 7	18 (3.6)	42 (8.4)	2.46 (1.39–4.33)	3.54 (1.74–7.20)
Normal	483 (96.4)	459 (91.6)	1.00	1.00
**APGAR score at 10 min**				
< 7	19 (3.8)	41 (8.2)	2.26 (1.29–3.95)	3.18 (1.58–6.41)
Normal	482 (96.2)	460 (91.8)	1.00	1.00
**LBW**				
Yes	33 (6.6)	31 (6.2)	0.94 (0.56–1.55)	0.92 (0.54–1.55)
No	468 (93.4)	470 (93.8)	1.00	1.00
**HBW**				
Yes	26 (5.2)	42 (8.4)	1.67 (1.01–2.77)	1.75 (1.05–2.92)
No	475 (94.8)	459 (91.6)	1.00	1.00
**Congenital disabilities**				
Yes	37 (7.4)	26 (5.2)	0.69 (0.41–1.15)	0.70 (0.41–1.17)
No	464 (92.6)	475 (94.8)	1.00	1.00
**At least one outcome** ^**#**^				
Yes	132 (26.3)	151 (30.1)	1.21 (0.92–1.59)	1.23 (0.93–1.64)
No	369 (73.7)	350 (69.9)	1.00	1.00

The prevalence of outcomes in the control group and acetaminophen use group were expressed as n (%)

Abbreviations: OR, odds ratio; CIs, confidence intervals; LBW, low birth weight; HBW, high birth weight

*Adjusted for maternal age, pre-pregnancy BMI, number of previous pregnancies, previous live births, history of adverse pregnancy outcomes, type of labour, infection with COVID−19, comorbidities, and co-medication

^#^Including stillbirth, miscarriage, preterm birth, APGAR score at 1 min, 5 min, and 10 min, LBW, HBW, and congenital disabilities

We conducted a stratified analysis of the drug exposure in different trimesters of pregnancy, as fetal growth and development characteristics in the three trimesters are different. Due to the limited sample size of the study cohort, we have pooled samples of acetaminophen use during the first and second trimesters to ensure that our analysis was as comprehensive as possible, allowing us to explore reliable conclusions. The results indicated that taking acetaminophen during the first and second trimesters of pregnancy could increase the odds of stillbirth (aOR = 5.04, 95% CI, 2.42–10.49), miscarriage (aOR = 5.43, 95% CI, 2.55–11.54), APGAR score < 7 at 1 min (aOR = 6.40, 95% CI,3.14–13.03), APGAR score < 7 at 5 min (aOR = 7.43, 95% CI, 3.41–16.21), and APGAR score < 7 at 10 min (aOR = 6.88, 95% CI, 3.19–14.84), and at least one outcome (aOR = 1.78, 95% CI, 1.19–2.65) (Table 3). However, exposure to acetaminophen in the third trimester only increased the odds of HBW (aOR = 1.89, 95% CI, 1.09–3.27) (Supplementary Table 1).

**Table 3:** Exposure to acetaminophen during first and second trimesters of pregnancy and risk of adverse perinatal outcomes in comparison with control group

Outcomes	Control (*n* = 501) n (%)	Acetaminophen use during first and second trimesters of pregnancy (*n* = 169) n (%)	Crude OR (95% CI)	*Adjusted OR (95% CI)
**Stillbirth**				
Yes	24 (4.8)	36 (21.3)	5.38(3.10–9.34)	5.04(2.42–10.49)
No	477 (95.2)	133 (78.7)	1.00	1.00
**Miscarriage**				
Yes	24 (4.8)	36 (21.3)	5.38(3.10–9.34)	5.43(2.55–11.54)
No	477 (95.2)	133 (78.7)	1.00	1.00
**Preterm birth**				
Yes	42 (8.4)	7 (4.1)	0.47(0.21–1.07)	0.57(0.24–1.34)
No	459 (91.6)	162 (95.9)	1.00	1.00
**APGAR score at 1 min**				
< 7	20 (4.0)	36 (21.3)	6.51(3.65–11.62)	6.40(3.14–13.03)
Normal	481 (96.0)	133 (78.7)	1.00	1.00
**APGAR score at 5 min**				
< 7	18 (3.6)	36 (21.3)	7.26(4.00−13.20)	7.43(3.41–16.21)
Normal	483 (96.4)	133 (78.7)	1.00	1.00
**APGAR score at 10 min**				
< 7	19 (3.8)	36 (21.3)	6.87(3.81–12.36)	6.88(3.19–14.84)
Normal	482 (96.2)	133 (78.7)	1.00	1.00
**LBW**				
Yes	33 (6.6)	8 (4.7)	0.71(0.32–1.56)	0.94(0.41–2.18)
No	468 (93.4)	161 (95.3)	1.00	1.00
**HBW**				
Yes	26 (5.2)	10 (5.9)	1.15(0.54–2.44)	1.42(0.65–3.09)
No	475 (94.8)	159 (94.1)	1.00	1.00
**Birth defects**				
Yes	37 (7.4)	12 (7.1)	0.96(0.49–1.88)	1.02(0.50–2.06)
No	464 (92.6)	157 (92.9)	1.00	1.00
**At least one outcome** ^**#**^				
Yes	132 (26.3)	64 (37.9)	1.70(1.18–2.46)	1.78(1.19–2.65)
No	369 (73.7)	105 (62.1)	1.00	1.00

The prevalence of outcomes in the control group and acetaminophen use group were expressed as n (%)

Abbreviations: OR, odds ratio; CIs, confidence intervals; LBW, low birth weight; HBW, high birth weight

*Adjusted for maternal age, pre-pregnancy BMI, number of previous pregnancies, previous live births, history of adverse pregnancy outcomes, type of labour, infection with COVID−19, comorbidities, and co-medication

^#^Including stillbirth, miscarriage, preterm birth, APGAR score at 1 min, 5 min, and 10 min, LBW, HBW, and congenital disabilities

As the accumulation of acetaminophen may be harmful to both the fetal and maternal liver and potentially lead to adverse perinatal outcomes, we have conducted a comprehensive investigation to estimate the association between the frequency of acetaminophen use during pregnancy and adverse perinatal outcomes. Pregnant women who used acetaminophen once a day had increased odds of stillbirth (aOR = 2.24, 95% CI, 1.14–4.40), APGAR score < 7 at 1 min (aOR = 3.08, 95% CI, 1.60–5.92), APGAR score < 7 at 5 min (aOR = 3.46, 95% CI, 1.67–7.15), APGAR score < 7 at 10 min (aOR = 3.09, 95% CI, 1.51–6.34), and HBW (aOR = 1.79, 95% CI, 1.06–3.03) compared with control group. Using acetaminophen more than twice a day was significantly associated with preterm birth (aOR = 2.40, 95% CI, 1.12–5.18), APGAR score < 7 at 1 min (aOR = 5.22, 95% CI, 1.68–16.25), APGAR score < 7 at 5 min (aOR = 4.23, 95% CI,1.08–16.62), and APGAR score < 7 at 10 min (aOR = 3.97, 95% CI, 1.01–15.57) (Table 4).

**Table 4:** Frequency of acetaminophen use during pregnancy and risk of adverse perinatal outcomes in comparison with unexposed

Outcomes	Control (*n* = 501) n (%)	Acetaminophen use (once a day) (*n* = 437) n (%)	Crude OR (95% CI)	*Adjusted OR (95% CI)	Acetaminophen use (more than twice a day) (*n* = 64) n (%)	Crude OR (95% CI)	*Adjusted OR (95% CI)
**Stillbirth**							
Yes	24 (4.8)	36 (8.2)	1.78(1.05–3.04)	2.24(1.14–4.40)	5 (7.8)	1.68(0.62–4.58)	2.82(0.71–11.26)
No	477 (95.2)	401 (91.8)			59 (92.2)		
**Miscarriage**							
Yes	24 (4.8)	32 (7.3)	1.57(0.91–2.71)	1.82(0.91–3.64)	4 (6.2)	1.33(0.45–3.95)	1.86(0.43–8.13)
No	477 (95.2)	405 (92.7)			60 (93.8)		
**Preterm birth**							
Yes	42 (8.4)	35 (8.0)	0.95(0.60–1.52)	0.89(0.55–1.45)	12 (18.7)	2.52(1.25–5.09)	2.40(1.12–5.18)
No	459 (91.6)	402 (92.0)			52 (81.3)		
**APGAR score at 1 min**							
< 7	20 (4.0)	40 (9.2)	2.42(1.39–4.21)	3.08(1.60–5.92)	7 (10.9)	2.95(1.20–7.29)	5.22(1.68–16.25)
Normal	481 (96.0)	397 (90.8)			57 (89.1)		
**APGAR score at 5 min**							
< 7	18 (3.6)	37 (8.5)	2.48(1.39–4.43)	3.46(1.67–7.15)	5 (7.8)	2.27(0.81–6.35)	4.23(1.08–16.62)
Normal	483 (96.4)	400 (91.5)			59 (92.2)		
**APGAR score at 10 min**							
< 7	19 (3.8)	36 (8.2)	2.28(1.29–4.03)	3.09(1.51–6.34)	5 (7.8)	2.15(0.77–5.97)	3.97(1.01–15.57)
Normal	482 (96.2)	401 (91.8)			59 (92.2)		
**LBW**							
Yes	33 (6.6)	26 (5.9)	0.90(0.53–1.53)	0.87(0.50–1.51)	5 (7.8)	1.20(0.45–3.20)	1.25(0.44–3.58)
No	468 (93.4)	411 (94.1)			59 (92.2)		
**HBW**							
Yes	26 (5.2)	37 (8.5)	1.69(1.01–2.84)	1.79(1.06–3.03)	5 (7.8)	1.55(0.57–4.19)	1.52(0.54–4.26)
No	475 (94.8)	400 (91.5)			59 (92.2)		
**Birth defects**							
Yes	37 (7.4)	24 (5.5)	0.73(0.43–1.24)	0.75(0.44–1.28)	2 (3.1)	0.41(0.10–1.72)	0.38(0.087–1.69)
No	464 (92.6)	413 (94.5)			62 (96.9)		
**At least one outcome** ^**#**^							
Yes	132 (26.3)	129 (29.5)	1.17(0.88–1.56)	1.19(0.89–1.61)	22 (34.4)	1.46(0.84–2.55)	1.54(0.85–2.78)
No	369 (73.7)	308 (70.5)			42 (65.6)		

The prevalence of outcomes in the control group and acetaminophen use group were expressed as n (%)

Abbreviations: OR, odds ratio; CIs, confidence intervals; LBW, low birth weight; HBW, high birth weight

*Adjusted for maternal age, pre-pregnancy BMI, number of previous pregnancies, previous live births, history of adverse pregnancy outcomes, type of labour, infection with COVID−19, comorbidities, and co-medication

^#^Including stillbirth, miscarriage, preterm birth, APGAR score at 1 min, 5 min, and 10 min, LBW, HBW, and congenital disabilities

## Discussion

### Main findings

The purpose of our study was to assess the association between acetaminophen use during pregnancy and adverse pregnancy outcomes. Our results showed potential risks associated with acetaminophen use during pregnancy. Furthermore, different trimesters of pregnancy and doses of drug use were also found to increase the risks of adverse pregnancy outcomes. These findings are expected to provide valuable advice to healthcare providers for the development of drug management measures to promote safe use during pregnancy.

In contrast to recently published studies [14–16], our study found an association between acetaminophen exposure during pregnancy and stillbirth, preterm birth, miscarriage, and HBW. Besides, our research shows that taking acetaminophen during the first and second trimesters of pregnancy was associated with a significantly higher risk of adverse perinatal outcomes than exposure in the third trimester. However, previous studies reported that exposure to acetaminophen during the first and second trimesters of pregnancy did not increase the risk of adverse perinatal outcomes [17–19]. There are several reasons why the results of our study may differ from those of previous studies. First, We gathered information on drug use in pregnant women from medical records, while other studies relied on patient self-reports. So, we might exaggerate or underestimate drug use in pregnant women outside the hospital. Secondly, the confounding factors in different studies differed, leading to inconsistency in the final analysis results. In addition, compared with other studies, our sample size was limited. Therefore, the research results need to be further confirmed.

Our findings suggested that exposure to acetaminophen during pregnancy might increase the risk of stillbirth, which was consistent with results from a previously published cohort study [5]. Although the current mechanism was not precise, relevant studies suggested that the role of acetaminophen in the inhibition of prostaglandin signaling might potentially result in the constriction of the ductus arteriosus, which could ultimately lead to fetal loss or life-threatening cardiac failure in the newborn [20]. Results from an animal study indicated that prenatal exposure to acetaminophen interferes with maternal immune and endocrine adaptation to pregnancy, affects placental function, and impairs fetal maturity and immune development [21]. Evidence revealed that placental dysfunction may be a contributing factor in many cases of unexplained stillbirth [22]. Stillbirth is a complex phenomenon, and several factors have been identified as potential risk factors, such as nulliparity, advanced age, smoking, obesity, race, prenatal care, and so on [22]. Hence, further research must identify and exclude relevant confounding factors and establish more conclusive evidence.

Another important conclusion is that exposure to acetaminophen during pregnancy increases the risk of APGAR score < 7, which a previous study has proved [5]. APGAR score < 7 should be taken into account because neonatal Apgar scores were a widely used method for quickly reporting the health status of a newborn after birth and predicting the need for resuscitation. Low Apgar scores (< 7) measured at 5 min have been associated with short-term adverse outcomes, such as higher infant mortality and increased hospital admissions, and long-term adverse effects, like abnormal neurological development, including language, hearing, speech, and psychological development [23, 24].

Upon stratifying the data according to the frequency of drug usage, we observed a significant increase in the incidence of preterm birth among those who had consumed drugs more than two times daily, which was in contrast to prior studies [4, 14, 15]. Acetaminophen is a prostaglandin inhibitor, and the related studies reported that the effect of prostaglandin on tissue targets is influenced by the prostaglandin receptor, where PGE2 and PGI2 have been shown to cause vascular smooth muscle relaxation and vasodilation in many circumstances [25]. However, since our conclusion contradicted most of the existing findings and the limitation of the small sample size, the current research conclusions must be treated cautiously.

### Strengths and limitations

An essential strength of this study was minimizing the interference of potential confounders on the results and increasing the reliability of the results through the use of propensity score matching. Secondly, we conducted stratified analyses based on different trimesters of pregnancy, which helped identify potential danger signals for acetaminophen exposure during specific periods. Furthermore, we considered the dose-response relationship of the drug and controlled for confounding by indications.

Our findings should be considered in light of the limitations. Medication information for pregnant women was based on EMR, which might underestimate the acetaminophen dosage, frequency, and duration used during pregnancy. Secondly, although we included relevant confounding factors to make adjustments based on previous studies and clinical experience, we could not exclude the influence of other confounding factors such as ethnicity, education, family income, smoking, and alcohol due to the lack of data. In addition, due to the limitation of sample size, our results may be biased. Finally, this was a single-center study carried out with a modest sample size, limiting our findings’ generality.

## Conclusions

Our research indicated that using acetaminophen during pregnancy could lead to adverse perinatal outcomes, particularly in the first and second trimesters. In addition, the frequency of acetaminophen use may be an essential risk factor for the occurrence of risk. However, our study has some methodological and sample size limitations, so future studies are needed to provide more reliable evidence for supporting the safety of drug use among pregnant women.

### Electronic supplementary material

Below is the link to the electronic supplementary material.

Supplementary Material 1

## Data Availability

Data is provided within the manuscript or supplementary information files.

## Abbreviations

aOR: Adjusted OR
HBW: High birth weight
EMR: Electronic medical record
NSAIDs: Non-steroidal anti-inflammatory drugs
LBW: Low birth weight
BMI: Body Mass Index
SD: Standard deviation
IQR: Interquartile range
CIs: Confidence intervals
