# Supplementary material for: Association of acetaminophen use with perinatal outcomes among pregnant women: a retrospective cohort study with propensity score matching
Source: BMC Pregnancy Childbirth. 2024 Apr 11;24:268. doi: 10.1186/s12884-024-06480-5 (PMC11010302; doi:10.1186/s12884-024-06480-5)
Supplement: Supplementary file 1 — Supplementary Material 1 [file 12884_2024_6480_MOESM1_ESM.docx]

**Supplementary Table 1** Exposure to acetaminophen during third trimester of pregnancy and risk of adverse perinatal outcomes in comparison with control group

| **Outcomes** | **Control (n=501) n (%)** | **Acetaminophen use during third trimester of pregnancy (n=332) n (%)** | **Crude OR (95% CI)** | ***Adjusted** **OR (95% CI)** |
| --- | --- | --- | --- | --- |
| **Stillbirth** |  |  |  |  |
| Yes | 24 (4.8) | 5 (1.5) | 0.30(0.12-0.81) | 0.57(0.20-1.65) |
| No | 477 (95.2) | 327 (98.5) |  |  |
| **Preterm birth** |  |  |  |  |
| Yes | 42 (8.4) | 40 (12.0) | 1.50(0.95-2.37) | 1.27(0.79-2.05) |
| No | 459 (91.6) | 292 (88.0) |  |  |
| **APGAR score at 1 min** |  |  |  |  |
| <7 | 20 (4.0) | 11 (3.3) | 0.82(0.39-1.74) | 1.40(0.61-3.20) |
| Normal | 481 (96.0) | 321 (96.7) |  |  |
| **APGAR score at 5 min** |  |  |  |  |
| <7 | 18 (3.6) | 6 (1.8) | 0.49(0.19-1.26) | 1.00(0.36-2.82) |
| Normal | 483 (96.4) | 326 (98.2) |  |  |
| **APGAR score at 10 min** |  |  |  |  |
| <7 | 19 (3.8) | 5 (1.5) | 0.39(0.14-1.05) | 0.79(0.27-2.33) |
| Normal | 482 (96.2) | 327 (98.5) |  |  |
| **LBW** |  |  |  |  |
| Yes | 33 (6.6) | 23 (6.9) | 1.06(0.61-1.83) | 0.91(0.51-1.61) |
| No | 468 (93.4) | 309 (93.1) |  |  |
| **HBW** |  |  |  |  |
| Yes | 26 (5.2) | 32 (9.6) | 1.95(1.14-3.34) | 1.89(1.09-3.27) |
| No | 475 (94.8) | 300 (90.4) |  |  |
| **Birth defects** |  |  |  |  |
| Yes | 37 (7.4) | 14 (4.2) | 0.55(0.29-1.04) | 0.55(0.29-1.04) |
| No | 464 (92.6) | 318 (95.8) |  |  |
| **At least one outcome^#^** |  |  |  |  |
| Yes | 132 (26.3) | 87 (26.2) | 0.99(0.72-1.36) | 1.02(0.74-1.41) |
| No | 369 (73.7) | 245 (73.8) |  |  |

The prevalence of outcomes in the control group and acetaminophen use group were expressed as n (%).

Abbreviations: OR, odds ratio; CIs, confidence intervals; LBW, low birth weight; HBW, high birth weight.

*Adjusted for maternal age, pre-pregnancy BMI, number of previous pregnancies, previous live births, history of adverse pregnancy outcomes, type of labour, infection with COVID‐19, comorbidities, and co-medication.

^#^Including stillbirth, miscarriage, preterm birth, APGAR score at 1 min, 5 min, and 10 min, LBW, HBW, and congenital disabilities.
